# Estimates of the Proportion of Older White Men Who Would Be Recommended for Pharmacologic Treatment by the New US National Osteoporosis Foundation Guidelines

**DOI:** 10.1002/jbmr.55

**Published:** 2010-02-04

**Authors:** Meghan G Donaldson, Peggy M Cawthon, Lily Y Lui, John T Schousboe, Kristine E Ensrud, Brent C Taylor, Jane A Cauley, Teresa A Hillier, Thuy T Dam, Jeff R Curtis, Dennis M Black, Douglas C Bauer, Eric S Orwoll, Steven R Cummings

**Affiliations:** 1 San Francisco Coordinating Center, California Pacific Medical CenterSan Francisco, CA, USA; 2 Park Nicollet Health Services and Division of Health Policy and Management, University of MinnesotaMinneapolis, MN, USA; 3 University of Minnesota and CCDOR VA Medical Center, Department of MedicineMinneapolis, MN, USA; 4 University of PittsburghPittsburgh PA, USA; 5 Kaiser Permanente Center for Health ResearchPortland, OR, USA; 6 Division of Geriatrics and Aging, Columbia UniversityNew York, NY, USA; 7 University of Alabama at Birmingham, Division of Clinical Immunology and RheumatologyBirmingham, AL, USA; 8 University of California San Francisco, Department of Epidemiology and BiostatisticsSan Francisco, CA, USA; 9 Oregon Health and Science University, Department of MedicinePortland, OR, USA

**Keywords:** osteoporosis, men, treatment, guidelines, epidemiology, bone density

## Abstract

The new US National Osteoporosis Foundation's (NOF's) *Clinician's Guide to Prevention and Treatment of Osteoporosis* includes criteria for recommending pharmacologic treatment based on history of hip or vertebral fracture, femoral neck or spine bone mineral density (BMD) *T*-scores of −2.5 or less, and presence of low bone mass at the femoral neck or spine plus a 10-year risk of hip fracture of 3% or greater or of major osteoporotic fracture of 20% or greater. The proportion of men who would be recommended for treatment by these guidelines is not known. We applied the NOF criteria for treatment to men participating in the Osteoporotic Fractures in Men Study (MrOS). To determine how the MrOS population differs from the general US population of Caucasian men aged 65 years and older, we compared men in MrOS with men who participated in the National Health and Nutrition Examination Survey (NHANES) III on criteria included in the NOF treatment guidelines that were common to both cohorts. Compared with NHANES III, men in MrOS had higher femoral neck BMD values. Application of NOF guidelines to MrOS data estimated that at least 34% of US white men aged 65 years and older and 49% of those aged 75 years and older would be recommended for drug treatment. Application of the new NOF guidelines would result in recommending a very large proportion of white men in the United States for pharmacologic treatment of osteoporosis, for many of whom the efficacy of treatment is unknown. © 2010 American Society for Bone and Mineral Research.

## Introduction

The US National Osteoporosis Foundation (NOF) recently released new guidelines, *Clinician's Guide to Prevention and Treatment of Osteoporosis*, that include comprehensive information about how to prevent fractures and mitigate their consequences.(1) The guidelines include recommendations about pharmacologic treatment to prevent fractures (Table 1). They recommend treatment based on history of hip or vertebral fracture, hip and spine bone mineral density (BMD) *T*-scores, and—among those with low bone mass—an elevated 10-year risk of fracture estimated from the World Health Organization (WHO) risk index (FRAX).(2)

**Table 1:** Criteria for Recommending Pharmacologic Treatment from the US NOF Guidelines

In women and men aged 50 years and older, pharmacologic therapy should be recommended for those with any one of the following:
• A history of hip fracture or clinical or radiographic spine fracture
• *T*-score of −2.5 or less at femoral neck or spine^a^
• Low bone mass (osteopenia), *T*-scores of −1.0 to −2.5 at the femoral neck or lumbar spine and any of the following:
•≥3% 10-year probability of hip fracture *or*
•≥20% 10-year probability of a major osteoporotic fracture based on the WHO model for the United States

a After excluding secondary causes.

Two approaches to treatment may be considered: one based on BMD alone and the other based on absolute risk of fracture. Although BMD is a strong predictor of hip and nonvertebral fractures in men,(3) over three-quarters of all fractures occur in men without WHO BMD-defined osteoporosis.(4) Thus treatment that is initiated based on BMD alone will not treat a large proportion of men who eventually go on to fracture. In general, compared with treatment based on BMD alone, the emphasis on treating based on absolute risk of fracture will decrease the proportion of 50- to 60-year-old men who are treated with drugs because they have a low risk of fractures. On the other hand, this approach may result in treating a large proportion of older people, particularly older white men who have a higher absolute risk of fracture. The proportions of various age groups that would be treated under the new NOF guidelines are not known. We previously estimated the proportion of older white women who participated in the Study of Osteoporotic Fractures that would be treated under the new NOF guidelines,(5) and we aim to extend these findings to men. Therefore, we used data from the Osteoporotic Fractures in Men Study (MrOS) and applied the new NOF treatment guidelines to estimate the proportion of white men aged 65 years and older who would be recommended for pharmacologic treatment. MrOS is a community-based sample of men from six communities. To confirm how well MrOS represents the US white male population aged 65 years and older, we compared the MrOS population with white males aged 65 years and older who participated in the third National Health and Nutrition Examination Survey (NHANES III) on treatment criteria listed in the NOF guidelines (including FRAX) common to both cohorts. This was done to gauge the similarities of the two cohorts on treatment criteria and to assess the generalizability of the proportion that would be recommended for treatment in the US white male population compared with MrOS. Since NHANES III was missing several components required to calculate FRAX, it was not possible to directly compare the proportion recommended for treatment between the NHANES III cohort and the MrOS cohort.

## Methods

We used data from the Osteoporotic Fractures in Men Study (MrOS), a prospective study of community-dwelling men aged 65 years and older recruited from six communities in the United States: Birmingham, Alabama, Minneapolis, Minnesota, Palo Alto, California, Pittsburgh Pennsylvania, Portland, Oregon, and San Diego, California.(6,7) Participants were recruited from population-based listings and mass mailings between 2000 and 2002. Men were not recruited on the basis of any risk factors for osteoporosis. All participants provided informed consent. This study was approved by the Institutional Review Board at each of the participating sites.

### Demographic, anthropometric, lifestyle, and medical history

Baseline examinations took place from 2000 to 2002 (*n* = 5995). Men provided information regarding fracture history, smoking status, alcohol consumption, parental hip fracture history, rheumatoid arthritis, and corticosteroid use. All prescription medications recorded by the clinics were stored in an electronic medications inventory database (San Francisco Coordinating Center, San Francisco, CA, USA). Each medication was matched to its ingredient(s) based on the Iowa Drug Information Service (IDIS) Drug Vocabulary (College of Pharmacy, University of Iowa, Iowa City, IA, USA).

Height was measured with a wall-mounted Harpenden stadiometer (Holtain, Ltd., DyFed, United Kingdom). Weight was measured with a balance-beam scale, except for one of the clinical centers that used a digital scale (Portland). Body mass index (BMI) was calculated as weight in kilograms divided by height in meters squared.

### Bone mineral density

Bone mineral density (BMD) was obtained at the proximal femur and lumbar spine by dual-energy X-ray absorptiometry (DXA) using QDR 4500 densitometers (Hologic, Inc., Bedford, MA, USA). *T*-scores for the femoral neck (FN) and total hip were calculated based on the means and standard deviations for men obtained from the NHANES III.(8) *T*-scores for the spine were calculated using the reference value provided by the manufacturer.

### WHO 10-year absolute fracture risk

The WHO 10-year absolute risk of both hip fracture and major osteoporotic fracture (ie, hip, clinical spine, forearm, or shoulder) was calculated by the WHO Collaborating Centre for Metabolic Bone Disease in February 2009. Calculation of absolute risk was done following the FRAX algorithm.(2,9) FRAX is described in detail elsewhere. Briefly, the calculation of the 10-year probabilities is based on nine risk factors (ie, age, sex, BMI, previous history of fracture, parental history of hip fracture, current smoking, use of corticosteroids in past 3 months, presence of rheumatoid arthritis, and three or more alcoholic beverages per day). The 10-year probabilities for both hip and major osteoporotic fracture can be calculated with or without FN BMD. The NOF treatment guidelines use the 10-year absolute risks (of hip and major osteoporotic fractures) calculated using FN BMD.

### NHANES III

NHANES is a program of studies designed to assess the health and nutritional status of adults and children in the United States. The survey uses a stratified complex sampling strategy to identify and examine a nationally representative sample of approximately 5000 persons each year. We used data from white men aged 65 years and older who participated in NHANES III. A direct estimation of the proportion of white men aged 65 years and older who would be recommended for treatment under the NOF guidelines cannot be performed using NHANES III because lumbar spine BMD, paternal history of fracture, and personal history of fracture at skeletal sites other than the hip, spine, and wrist were not assessed in NHANES III. For those factors in FRAX and/or the NOF guidelines that were measured in both MrOS and NHANES III, we reported the mean values for continuous variables (ie, age, BMI, and BMD) and proportions for dichotomous variables (ie, personal history of fracture, maternal history of fracture, rheumatoid arthritis, current smoking, and consumption of three or more alcoholic drinks per day). NHANES III data were obtained from the publicly available data release (http://www.cdc.gov/nchs/nhanes.htm). Means and proportions were adjusted for the NHANES III sampling strategy, as recommended in the NHANES analysis guidelines, using the SURVEYMEANS and SURVEYFREQ procedures in SAS (SAS Institute, Inc., Cary, NC, USA).

### Analysis

Men in MrOS were excluded from the analysis if they were nonwhite. Although MrOS and NHANES III enrolled nonwhite men and FRAX probabilities can be calculated for several different ethnicities, the analysis was limited to white men because there were too few nonwhite men in the MrOS cohort to provide an accurate ethnicity-stratified analysis. Men also were excluded if they had missing data for any of the factors required to apply the NOF guidelines, including those required to calculate FRAX. We applied the NOF guidelines as given in Table 1 to determine the proportion of men who would be recommended for treatment.

Radiographic vertebral fracture status was not available in MrOS. However, this information is not collected routinely in the usual care setting, and therefore, in order to conservatively apply the guidelines, we did not include information about radiographic vertebral fractures in determining whether a man should be treated.

In the primary analysis, the criterion “past history of hip fracture or clinical or radiographic vertebral fracture” was conservatively limited to past history of hip fracture. We also estimated the proportion of men who would be recommended for treatment if a history of clinical vertebral fractures was included. In other secondary analyses, we applied the criterion “*T*-score of −2.5 or less at femoral neck or spine” and “osteopenia and 10-year risk of hip fracture of 3% ot greater or osteopenia and major osteoporotic fracture risk of 20% or greater” alone. Finally, to determine if the proportion of men who would be recommended for treatment differed by age, we analyzed men 65 years of age and older and 75 years of age and older.

## Results

A total of 5995 men participated in the baseline examination. Of these, 5362 self-identified as white. There were 3913 men who had complete data for the nine clinical risk factors in FRAX and femoral neck BMD. The two most common reasons for missing data were parental history of hip fracture and glucocorticoid use (*n* = 1438). An additional 11 men were missing one or more of the other FRAX variables. Finally, 26 men were missing spine BMD (an element of the NOF guidelines). Thus 3887 men were included in the analysis. Men who were missing at least one FRAX or NOF criteria had similar age, BMI, and femoral neck BMD to those included in the analysis.

Men in MrOS were slightly older (73.6 versus 72.2 years) and had slightly higher BMD values at the femoral neck than men 65 years of age and older who participated in NHANES III. Therefore, men in MrOS had a lower prevalence of *T*-scores below −2.5 and a higher number with *T*-scores above −1.0. Similar proportions of men in MrOS and NHANES reported a previous hip fracture compared with the men in NHANES (1.3%). A smaller proportion of men in MrOS reported consuming of three or more alcoholic beverages per day, and current smoking compared with that in NHANES (Table 2). The prevalence of bisphosphonate use among men in MrOS was 1.8%.

**Table 2:** Comparisons of Characteristics of White Men Aged 65 Years and Greater in NHANES III and in MrOS

Variable	NHANES III (*n* = 1101)	MrOS (*n* = 3887)
Age, years		
All, mean [95% confidence interval (CI)]	72.2 (71.9–72.6)	73.7 (73.5–73.8)
65 to 74 years, % (*n*)	69.5 (NA)	58.0 (2256)
≥75 years, % (*n*)	30.5 (NA)	42.0 (1631)
BMI, kg/m^2^		
All, mean (95% CI)	26.8 (26.5, 27.1)	27.4 (27.3, 27.5)
Bone Mineral Density		
Femoral neck, g/cm^2^		
All, mean (95% CI)	0.756 (0.746, 0.767)	0.782 (0.778, 0.786)
65-74, mean (95% CI)	0.775 (0.761, 0.789)	0.799 (0.795, 0.751)
≥75, mean (95% CI)	0.719 (0.704, 0.735)	0.757 (0.751, 0.763)
Femoral neck *T*-score		
All		
Normal,^a^ % (*n*)	35.2 (NA)	41.9 (1629)
Osteopenia (“low bone mass”), % (*n*)	53.9 (NA)	53.1 (2065)
Osteoporosis, % (*n*)	10.8 (NA)	5.0 (193)
Spine *T*-score		
All		
Normal,^a^ % (*n*)	NA	78.8 (3053)
Osteopenia (“low bone mass”), % (*n*)	NA	18.3 (711)
Osteoporosis, % (*n*)	NA	3.2 (123)
Fracture history (age ≥ 50 years)		
Hip		
All, % (*n*)	1.3 (NA)	1.3 (49)
65 to 74 years, % (*n*)	1.0 (NA)	0.7 (17)
≥75 years, % (*n*)	1.9 (NA)	2.0 (32)
Parental history of hip fracture		
Mother or father, % (*n*)	NA	17.2 (667)
Father only, % (*n*)	NA	3.9 (152)
Mother only, % (*n*)		
All, % (*n*)	9.5 (NA)	13.8 (535)
65 to 74 years, % (*n*)	10.5 (NA)	14.7 (332)
≥75 years, % (*n*)	7.7 (NA)	12.5 (203)
Rheumatoid arthritis		
All, % (*n*)	4.7 (NA)	4.7 (184)
≥3 alcoholic beverages/day		
All, % (*n*)	10.2 (NA)	3.8 (149)
Current smoker		
All, % (*n*)	13.7 (NA)	3.0 (116)

a Normal = *T*-score > −1.0; low bone mass = −1.0 ≥ *T*-score > −2.5; osteoporosis = *T*-score < −2.5.

Overall, applying the modified NOF guidelines to men aged 65 years and older in MrOS, 33.4% would be recommended for treatment (Table 3). When history of clinical vertebral fracture is included, 34.0% of men would be recommended for treatment. When the guidelines are applied to men aged 75 years and older, 49.1% would be recommended for treatment.

**Table 3:** NOF Criteria for Recommending Pharmacologic Therapy

	≥65 Years, % (*n*)	≥75 Years, % (*n*)
	*n* = 3887	*n* = 1631
Hip fracture	49 (1.3)	32 (2.0)
Clinical spine fracture	85 (2.2)	46 (2.8)
*T*-score ≤ −2.5 at		
Femoral neck	193 (5.0)	132 (8.1)
Spine	123 (3.2)	57 (3.5)
Femoral neck or spine	273 (7.0)	161 (9.9)
Low bone mass (osteopenia) at the femoral neck or spine and		
US FRAX 10-year probability of hip fracture ≥ 3%	1128 (29.0)	696 (42.7)
US FRAX 10-year probability of any major osteoporosis-related fracture ≥ 20%	322 (8.3)	187 (11.5)
Low bone mass (osteopenia) only at the femoral neck (not spine) and		
US FRAX 10-year probability of hip fracture ≥ 3%	1040 (26.8)	637 (39.1)
US FRAX 10-year probability of any major osteoporosis-related fracture ≥ 20%	279 (7.2)	157 (9.6)
All NOF criteria combined^a^	1329 (34.0)	800 (49.1)

a Using criteria: previous hip fracture, previous clinical spine fracture, osteoporosis (femoral neck or spine), low bone mass (femoral neck or spine), and both US FRAX 10-year probabilities.

Applying only the NOF guideline criterion of “*T*-score of −2.5 or less at the femoral neck or spine,” 7.0% of men would be recommended for drug treatment (Table 3). However, if *T*-score of −2.5 or less is limited to only the femoral neck, 5.0% would be recommended for treatment (Table 3). Applying only theNOF guideline criterion “low bone mass and 10-year risk of hip fracture of 3% or greater or major osteoporotic fracture risk of 20% or greater,” 29.2% of men would be recommended for drug treatment.

## Discussion

We estimate that application of the new NOF guidelines would result in recommending that pharmacologic treatment to prevent fractures should be initiated for about one-third of the participants in the MrOS study and approximately half the men over age 75. These figures may underestimate the proportions that would be recommended for treatment in the US population because the men in MrOS had somewhat higher BMD values than the men in the US population-based NHANES III survey. Furthermore, we did not include some criteria for treatment, such as a history or presence of a radiographic vertebral fracture, inclusion of which would further increase the number recommended for treatment.

Our estimates are consistent with results from the Canadian Multicenter Osteoporosis Study (CaMOS). In our study, we found that approximately 8% of men would be eligible for treatment using only the 10-year probability of major osteoporotic fracture of 20% or greater criterion. A similar analysis in the CaMOS cohort found that over 10% of white men aged 65 years or older would have a 10-year probability of fracture of 20% or greater based on BMD and the risk factors used in the WHO model.(10) Although the NOF cost-effectiveness analysis did not report the proportion of men who would be treated under the NOF guidelines, they do conclude that pharmacologic treatment would be cost-effective for the average 73-year-old white man.(11) Thus, given that the median age of men who participated in MrOS was 73 years, it is consistent that we estimated that a high proportion (34%) of men would be recommended for drug treatment.

Our analysis suggests that approximately one-third of the older white men recommended for pharmacologic treatment under the new guidelines would be included because they have low bone mass (osteopenia) at one of the two skeletal sites and a 10-year estimated probability of hip fracture of 3% or greater or a 10-year estimated probability of major osteoporotic fracture of 20% or greater. The FRAX probability of hip fracture risk (≥3%) was the criterion that would lead to the greatest number of men being treated (29% of total group). When so many people and such a large proportion of older men are recommended for drug treatment, it is important that the assumptions underlying the recommendations be based on robust data. In particular, it is important that there be strong evidence of substantial benefit from treatment among those with low bone mass and additional risk factors. To date, there have been no randomized, controlled trials testing the efficacy of antifracture drug treatment in this population.

We found that about 5% of men in MrOS had osteoporosis, defined as a femoral neck BMD *T*-score of −2.5 or less. A recent systematic review indicated that there were limited data to indicate that treatment was effective in reducing fractures in men.(12) Specifically, there have been two trials testing the efficacy of alendronate,(13,14) two of risedronate,(15,16) and two of parathyroid hormone(17,18) in men with osteoporosis. All studies demonstrated a significant increase in lumbar spine BMD, the primary endpoint, and three studies demonstrated a significant reduction in incident vertebral fractures(13–15) on active drug compared with placebo. However, the efficacy of these drugs to prevent both vertebral and, in particular, nonvertebral fractures remains unclear.

Our estimates have several limitations. First, MrOS is a cohort of community-dwelling volunteers and not a population-based sample. However, characteristics of the MrOS participants are similar to, or healthier than, those of the population-based NHANES III, and therefore may underestimate the proportion of men who would be recommended for treatment. Secondly, FRAX may underestimate the 10-year probability of major osteoporotic fracture among those who have a radiographic vertebral fracture. We conservatively excluded radiographic vertebral fractures when the FRAX 10-year probabilities were calculated by the WHO (i.e., radiographic vertebral fractures were not included as a “history of previous fracture” in the FRAX calculations). Finally, as this analysis from MrOS includes only white men age 65 or older this study is not able to estimate the proportion of younger men or men of other racial groups who would be recommended for pharmacologic treatment. Presumably, the proportion of people in these groups recommended for treated will be substantially less than the estimates from MrOS because they have a lower risk of fracture and lower prevalence of osteoporosis.

We conclude that the new NOF guidelines for pharmacologic treatment for osteoporosis would recommend drug therapy to at least one-third of white men aged 65 years and older and 50% of those older than 75 years of age. When such a large proportion of older men is recommended to receive drug treatment for osteoporosis, it is important that the assumptions that underlie that analysis be based on robust evidence. This would require a trial of bisphosphonate therapy and/or other pharmaceutical agents in women and men with just “low bone mass.”
